# Evaluation of large animal models for preclinical studies of heart failure with preserved ejection fraction using clinical score systems

**DOI:** 10.3389/fcvm.2023.1099453

**Published:** 2023-03-23

**Authors:** Ke Li, Cristiano Cardoso, Angel Moctezuma-Ramirez, Abdelmotagaly Elgalad, Emerson Perin

**Affiliations:** ^1^ Center for Preclinical Cardiovascular Research, The Texas Heart Institute, Houston, TX, United States; ^2^ Center for Clinical Research, The Texas Heart Institute, Houston, TX, United States

**Keywords:** large animal model, heart failure, HFPEF, HFA-PEFF, H2FPEF

## Abstract

Heart failure with preserved ejection fraction (HFpEF) is characterized by a complex, heterogeneous spectrum of pathologic features combined with average left ventricular volume and diastolic dysfunction. HFpEF is a significant public health problem associated with high morbidity and mortality rates. Currently, effective treatments for HFpEF represent the greatest unmet need in cardiovascular medicine. A lack of an efficient preclinical model has hampered the development of new devices and medications for HFpEF. Because large animal models have similar physiologic traits as humans and appropriate organ sizes, they are the best option for limiting practical constraints. HFpEF is a highly integrated, multiorgan, systemic disorder requiring a multipronged investigative approach. Here, we review the large animal models of HFpEF reported to date and describe the methods that have been used to create HFpEF, including surgery-induced pressure overloading, medicine-induced pressure overloading, and diet-induced metabolic syndrome. In addition, for the first time to our knowledge, we use two established clinical HFpEF algorithms (HFA-PEFF and H2FPEF scores) to evaluate the currently available large animal models. We also discuss new technologies, such as continuous remote pressure monitors and inflatable aortic cuffs, as well as how the models could be improved. Based on current progress and our own experience, we believe an efficient large animal model of HFpEF should simultaneously encompass multiple pathophysiologic factors, along with multiorgan dysfunction. This could be fully evaluated through available methods (imaging, blood work). Although many models have been studied, only a few studies completely meet clinical score standards. Therefore, it is critical to address the deficiencies of each model and incorporate novel techniques to establish a more reliable model, which will help facilitate the understanding of HFpEF mechanisms and the development of a treatment.

## 1. Introduction

Heart failure (HF) is a disease in which the heart loses its ability to provide sufficient forward output to meet the perfusion and oxygenation requirements of tissues while maintaining normal filling pressure (1). Heart failure with preserved ejection fraction (HFpEF) is characterized by normal left ventricular ejection fraction (LVEF, mostly defined as LVEF > 50%) and abnormal diastolic function, often with LV concentric remodeling or hypertrophy, but sometimes with normal ventricular geometry and LV volume (2–5). Approximately 50% of HF cases are HFpEF, which is associated with high morbidity and mortality rates (6). Although progress has been made recently using the SGLT-2 inhibitor to treat HFpEF (7, 8), overall, therapy for patients with HFpEF and their prognosis remain a challenge. This is in contrast to the latest breakthrough advances in treating HF with reduced ejection fraction (HFrEF) (9). Importantly, developing new therapies for HFpEF has been slow in part because of the absence of a reliable animal model (10). In this review, we highlight the progress to date in identifying an optimal large animal model for preclinical studies of HFpEF treatments. In addition, we discuss some promising options for the future.

## 2. Current understanding of the pathophysiology of HFpEF

**Figure 1:**
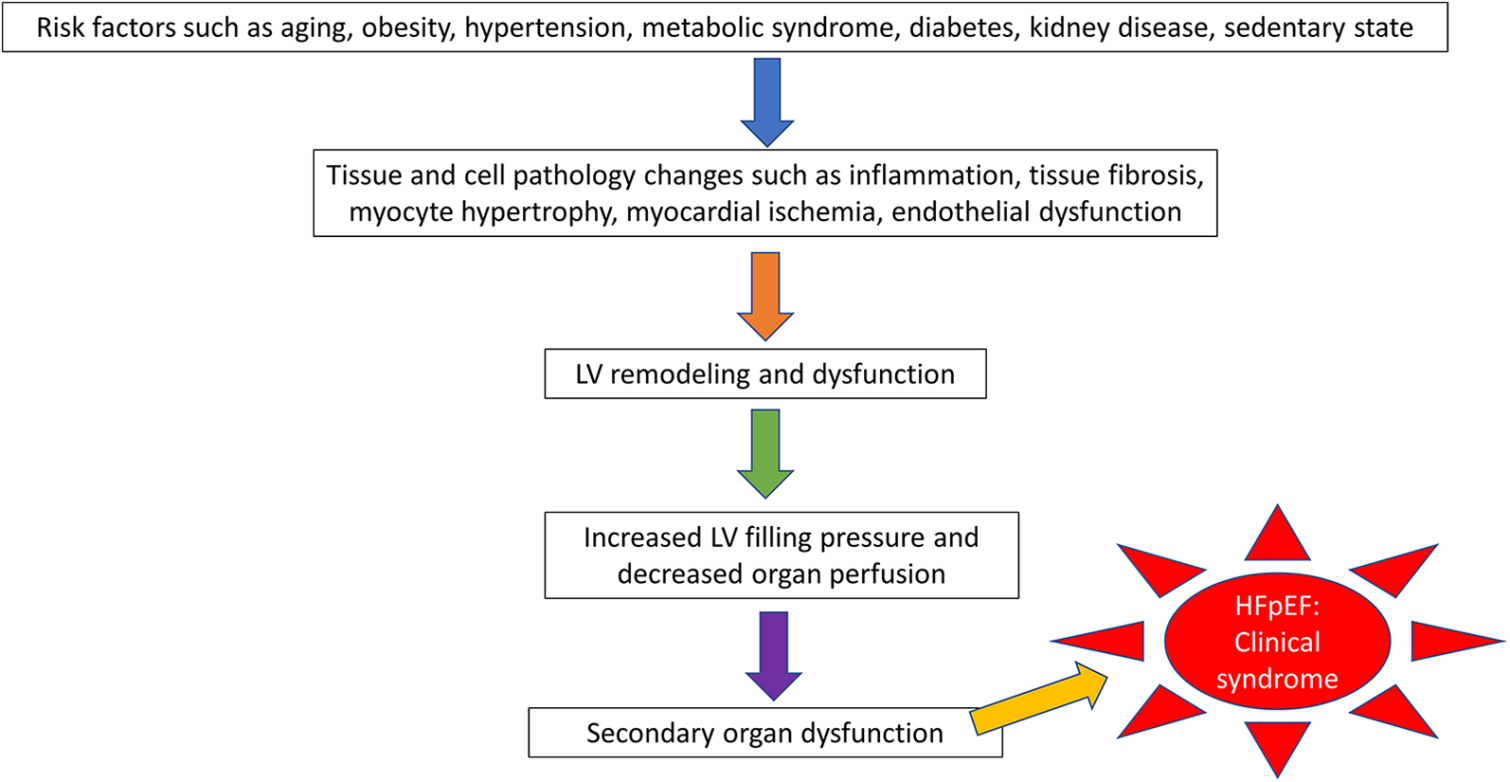
The pathophysiologic progression of heart failure with preserved ejection fraction (hFpEF).

Accurately understanding the pathophysiology of a disease is essential for developing an animal model. To date, the precise mechanism for the pathophysiology of HFpEF is incompletely understood. Unlike HFrEF, which largely results from ischemic heart disease or structural heart disease and is therefore easily translated into an animal model, HFpEF is a systemic condition with both cardiac and extra-cardiac features (9). Clinical risk factors for HFpEF include aging, obesity, metabolic syndrome, hypertension, sedentary state, coronary disease, and kidney disease, which all cause widespread tissue and cell injury through different mechanisms, such as systemic inflammation, tissue fibrosis, myocardial ischemia, myocyte hypertrophy, and abnormal energetics. These mechanisms further remodel LV structure and decrease LV function and hemodynamic status, finally leading to secondary organ dysfunction presenting with clinical symptoms (9, 11–15) (Figure 1).

In addition, recent data have shown an important role for the peripheral vascular system in HFpEF. Patients with HFpEF have abnormalities in the systemic vasculature, endothelium, adipocytes, and skeletal muscle (16, 17). Another important concept in the pathophysiology of HFpEF is the abnormal reserve (18). Even when baseline or resting function appears normal, cardiac, vascular, and peripheral reserve for coping with stressors is significantly diminished. Because HFpEF pathophysiology research is evolving quickly, this poses additional challenges to developing animal models that accurately represent human HFpEF.

## 3. Large animal models of HFpEF

Animal models are widely used to investigate the pathophysiology of HFpEF and to ultimately develop new treatments (19, 20). Because of the heterogeneous nature of HFpEF, designing an animal model in which all facets of HFpEF features are represented is difficult. However, some common features of HFpEF pathophysiology are less variable, such as LV diastolic function change (21), LV hypertrophy (22), and elevated natriuretic peptides (23). Several rodent models of HFpEF with diastolic dysfunction have contributed to our understanding of HFpEF mechanisms (24), but they are limited in that rodent hearts are not of comparable size, structure, or function to the hearts of humans. For device-based therapies in particular, large animal models are the only option for *in vivo* testing and are considered the last preclinical step before testing in humans. Therefore, large animal models of HFpEF are needed that can simulate HFpEF in humans. Currently, several types of large animal models of HFpEF are available that we will discuss here. To apply objective standards for evaluating the modeling of this complex clinical syndrome in large animals, we used clinical scores, as previously done in mice (24).

## 4. Clinical scores

**Figure 2:**
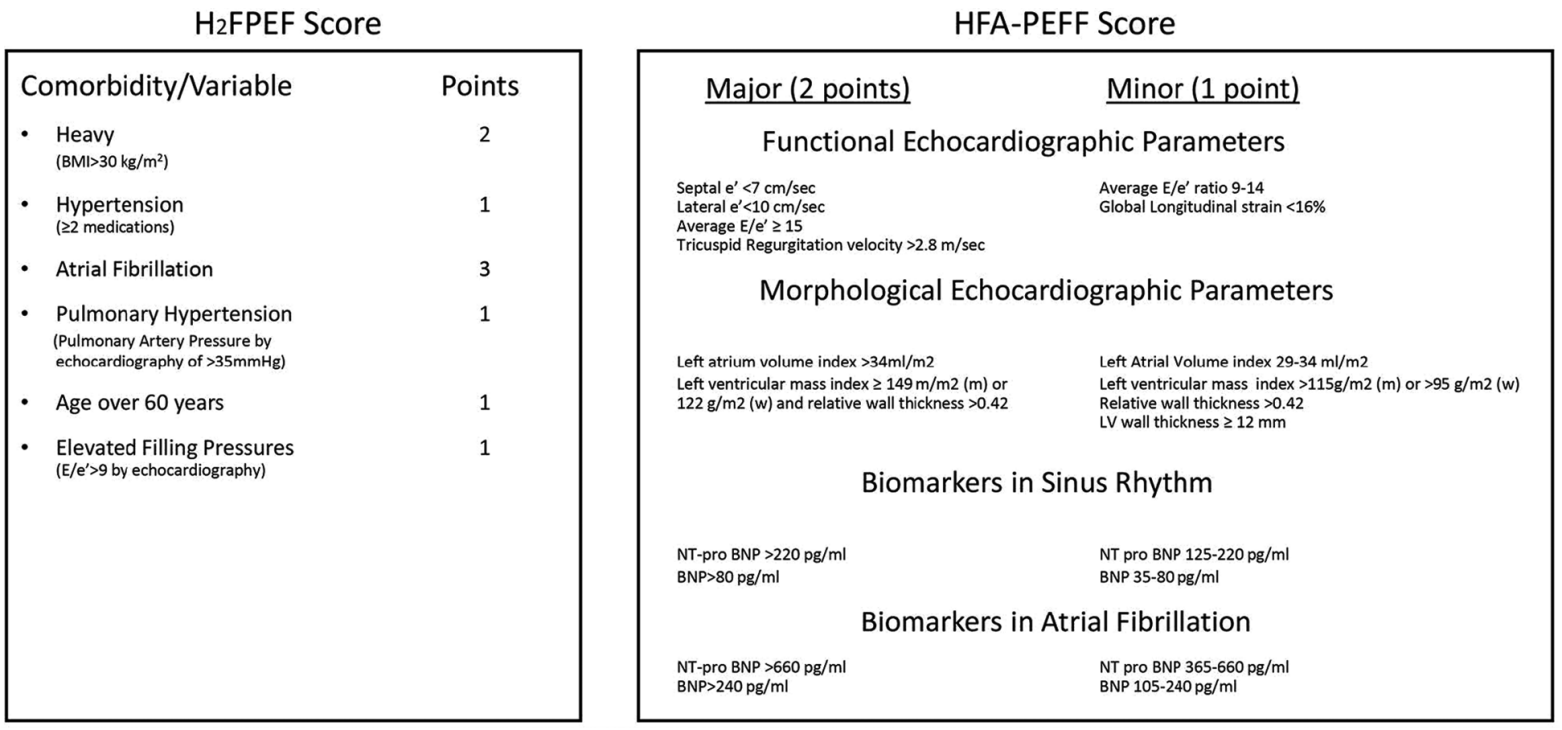
Illustration of the HFA-PEFF and H2FPEF score systems. From (27).

Two score systems have recently been used to diagnose HFpEF in patients: HFA-PEFF and H2FPEF (2, 25, 26) (Figure 2). Both HFpEF scores have been validated in various patient cohorts and community studies. Both HFpEF scores have been shown to categorize patients well, especially patients with intermediate and high scores (28–30). Despite some disadvantages of these score systems, such as an overly complicated diagnostic process (31) and misclassification in lower-score patients (32), they have been shown to have prognostic utility, suggesting that they capture key pathophysiologic components that determine outcomes in patients with HFpEF (33, 34).

### 4.1. Clinical diagnostic procedures

**Figure 3:**
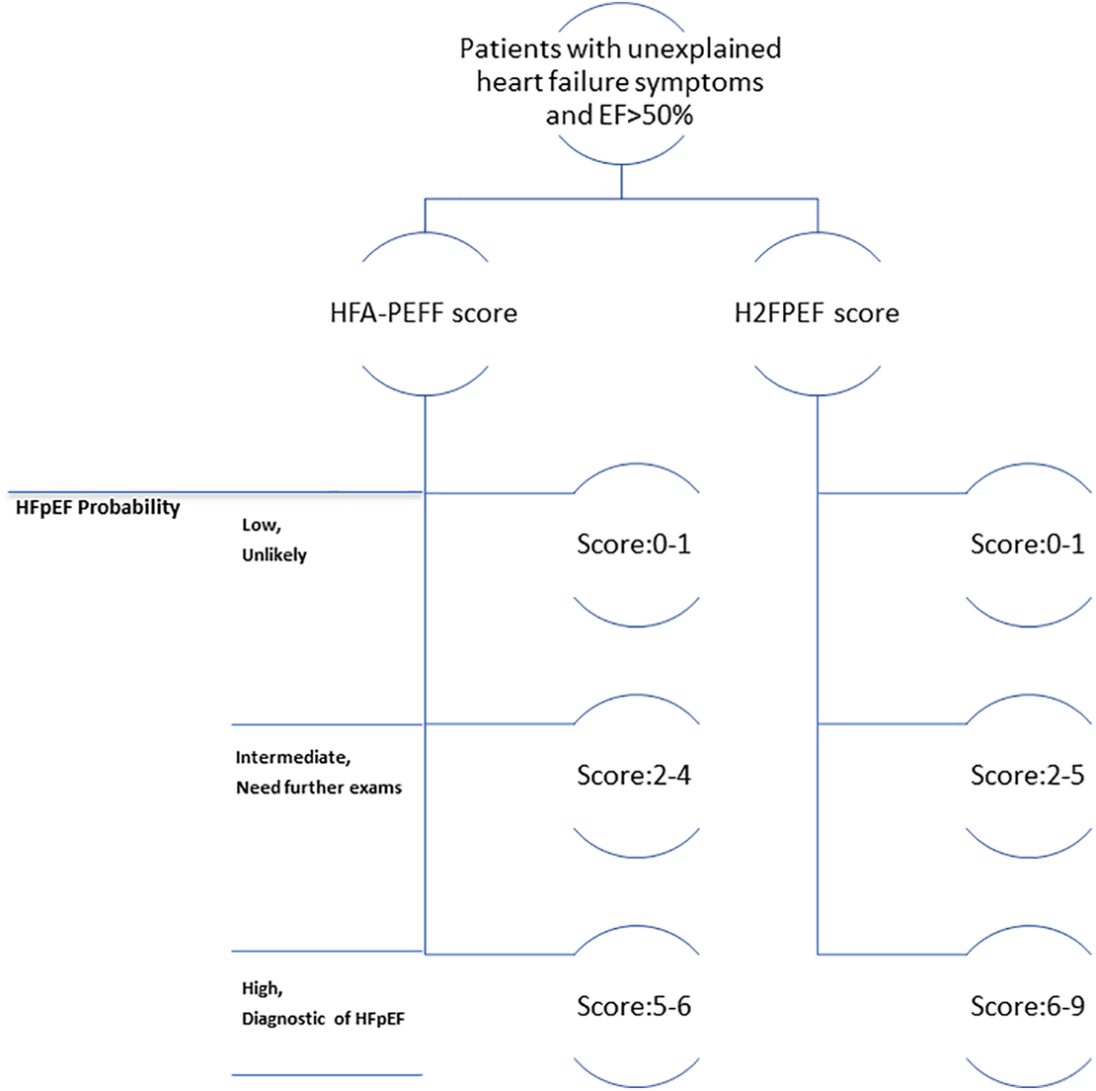
Clinical algorithm flowchart for diagnosing heart failure with preserved ejection fraction (hFpEF).

As a clinical syndrome, HFpEF is suspected when patients have symptoms or signs of HF with all three of the following features (5, 35) (Figure 3): 1) one or more symptoms of HF, such as dyspnea or fatigue, with or without physical signs of HF; 2) a LVEF ≥50%; and 3) no apparent cause of HF symptoms other than HFpEF. For patients who meet these criteria, it is suggested that the score systems be used for further diagnosis. Details of both score systems are provided in Figure 2. The probability of HFpEF is high when the HFA-PEFF score is 5 or 6, or the H2FPEF score is between 6 and 9. Both scores rely heavily on echocardiography measurements, especially the HFA-PEFF, which provides a direct reflection that cardiac factors, such as LV diastolic dysfunction, dominate the pathologic development of HFpEF. For the H2FPEF, multiple extra-cardiac risk factors are introduced, such as body mass and age.

### 4.2. Pretest of symptoms and signs of HF

LVEF should be maintained above 50%, which can be measured using transthoracic echocardiography, along with LV dimension and volume. However, because animals cannot verbalize, detecting symptoms in animals is not straightforward. As an alternative, we can use surrogate measurements that provide evidence of HF, such as blood natriuretic peptide level (also included in the HFA-PEFF score) and results of a stress or exercise test. Reduced exercise reserve caused by weakness or fatigue is a typical feature of HFpEF. If a model does not include any of these, it should not be regarded as a complete preclinical model.

### 4.3. Using the HFA-PEFF score

The HFA-PEFF score includes the following echocardiography measurements for LV function and morphology: LV wall thickness, LV end diastolic diameters, left atrial volume index, LV mass index, mitral inflow (E wave), tissue Doppler septal and lateral wall mitral velocity (e'), peak tricuspid regurgitation velocity (if applicable), and global longitudinal strain. In large animal studies, these parameters can be recorded by using transthoracic echocardiography and should be included for each animal. Therefore, an experienced echocardiographer is required.

### 4.4. Using the H2FPEF score

Similar to the HFA-PEFF score, the H2FPEF score includes LV diastolic function analysis, as well as the following extracardiac factors.

***Obesity.*** Body weight can be easily measured for a large animal every time the animal is sedated for an echocardiography procedure.

***Hypertension****.* In large animals, monitoring blood pressure without invasive procedures can be difficult. Recently, with the development of telemetry technology, some implantable devices have become available for use in large animals. For example, the implantable easyTel + (EMKA Technologies, Sterling, VA) device (36) can be used to continuously monitor blood pressure in large animals for up to 60 days.

***Age.*** Most large animals are enrolled in studies at a young age. For example, a pig will be considered “aging” when it reaches 13 to 15 years (37). Pigs this age are difficult to obtain from vendors, and keeping large animals until they reach senior age poses a huge financial burden. Thus, age is rarely considered in large animal studies.

The H2FPEF score also includes echocardiography parameters, such as pulmonary artery systolic pressure, Doppler mitral inflow (E wave), and tissue Doppler mitral velocity (e'), which should be acquired by using transthoracic echocardiography. For recording atrial fibrillation, a telemetry device for pigs is recommended that can be implanted into the pig's body and provide continuous electrocardiogram (EKG) monitoring (I-III, avL, avF, avR) (38). For cows and sheep that can be restrained in the stanchion, a regular EKG monitor can be used.

### 4.5. Gold standard

A major cardiac pathophysiologic change in patients with HFpEF is diastolic dysfunction. Therefore, hemodynamic assessment is the gold standard and may be an advantage for the verification of a model if included in a study. In large animals, hemodynamic measurements, including LV PV-loop, dp/dt, or tau index (39), can be performed by using catheters. In addition, catheters can be used to accurately evaluate pulmonary hypertension. Recently, researchers have used telemetry devices to monitor LV pressure continuously by implanting a pressure sensor into the left ventricle (36). However, per clinical score systems, such hemodynamic assessment is not required in most patients (2). We found that in many studies, hemodynamic measurements are the only method reported for evaluating diastolic function. Credit was still given to studies if they showed a significant diastolic function change.

## 5. Model validations with clinical scores

### 5.1. Aortic banding

**Figure 4:**
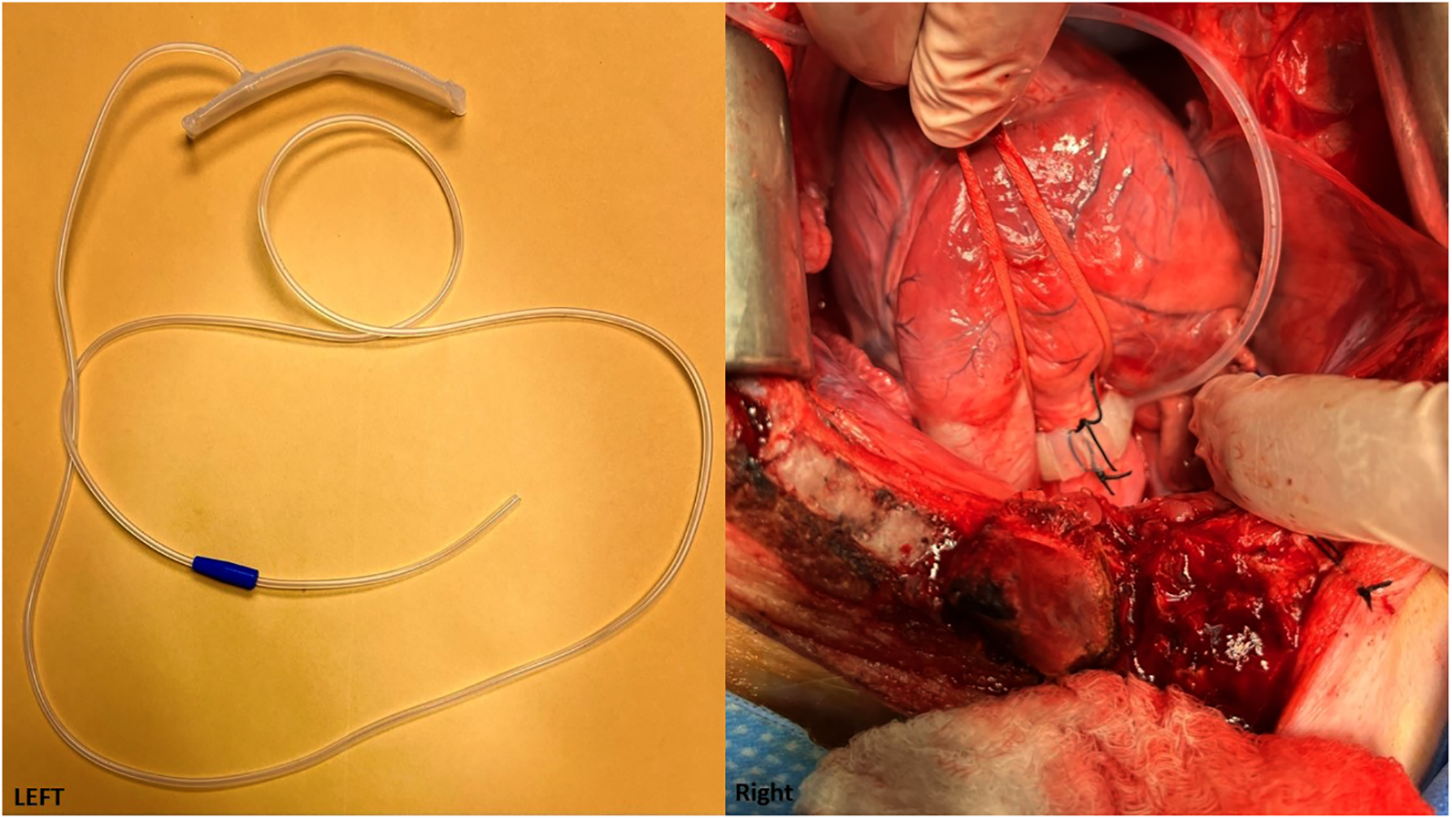
(Left) An adjustable aortic cuff that can be inflated to different pressure levels. (Cuff is from Access Technologies, Skokie, IL, www.norfolkaccess.com.) (Right) Surgical view showing the adjustable aortic cuff attached to the aortic root.

Aortic banding mimics high blood pressure by constricting the aorta, which in turn induces hypertension and further causes chronic LV pressure overload and hypertrophy. This model is primarily used in pigs. Constrictors can be placed in the aortic root, ascending aorta, or descending aorta. In these studies, LV fibrosis is consistently documented as the major pathologic change. The two types of constrictors are the fixed-size or pressure constrictor (40–49), or the inflatable cuff (50, 51) (Figure 4) that can be used to adjust the size and pressure gradually over time through a subcutaneous tunnel that can be connected externally.

Currently, aortic banding is the most widespread, well-known method for inducing HFpEF. However, in the 12 studies we reviewed, only 4 studies (44–46, 50) were qualified under HFA-PEFF, and none of them were qualified under H2FPEF (Table 1). Among all studies, only the feline model created by Wallner et al. (46) and the pig model by Tan et al. (45) provided thorough echocardiographic evaluation for both diastolic function (including E/e') and morphology change. All other studies focused on the LV morphology changes only, such as wall thickness and left ventricular mass, although a few of them provided hemodynamic analysis. However, not all hemodynamic analyses showed diastolic function change (43). In addition, only a small portion of studies showed brain natriuretic peptide (BNP) results. Therefore, only 25% studies were qualified as an HFpEF model on the basis of the HFA-PEFF score. The H2FPEF score was even worse, with 4 as the highest score. Only one study successfully demonstrated increased weight, in addition to hypertension, in the experimental group (50), and only 2 studies (45, 46) showed both pulmonary hypertension and diastolic function change.

**Table 1:** Clinical scores of aortic banding models.

		HFA-PEFF				H2FPEF								
Article	Species	Function	Morphology	BNP	Total	Obesity	HT	Age	AFib	PH	DD	Total	Gold standard	EF preserve
Ye et al. (49)	Pig	0	2	0	2	0	1	0	0	0	0	1	NA	NA
Gong et al. (41)	Pig	0	2	0	2	0	1	0	0	0	0	1	NA	NA
Wang et al. (47)	Pig	0	2	0	2	0	1	0	0	0	0	1	NA	NA
Marshall et al. (44)	Pig	2	2	2	6	0	1	0	0	0	1	2	Yes	Yes
Hiemstra et al. (42)	Pig	0	2	0	2	0	1	0	0	0	0	1	NA	Yes
Emter et al. (40)	Pig	0	2	0	2	0	1	0	0	0	0	1	NA	Yes
Xiong et al. (48)	Pig	0	2	0	2	0	1	0	0	0	0	1	NA	Yes
Ishikawa et al. (43)	Pig	0	2	0	2	0	1	0	0	0	0	1	Yes	Yes
Wallner et al. (46)	Feline	2	2	2	6	0	1	0	0	1	1	3	Yes	Yes
Tan et al. (45)	Pig	2	2	2	6	0	1	0	0	1	1	3	No	Yes
Yarbrough et al. (51)	Pig	2	2	0	4	0	1	0	0	0	1	2	Yes	Yes
Charles et al. (50)	Pig	2	2	2	6	2	1	0	0	0	1	4	Yes	Yes

AFib, atrial fibrillation; BNP, brain natriuretic peptide; DD, diastolic dysfunction; HT, hypertension; NA, not applicable; PH, pulmonary hypertension.

### 5.2. Aortic stent

**Figure 5:**
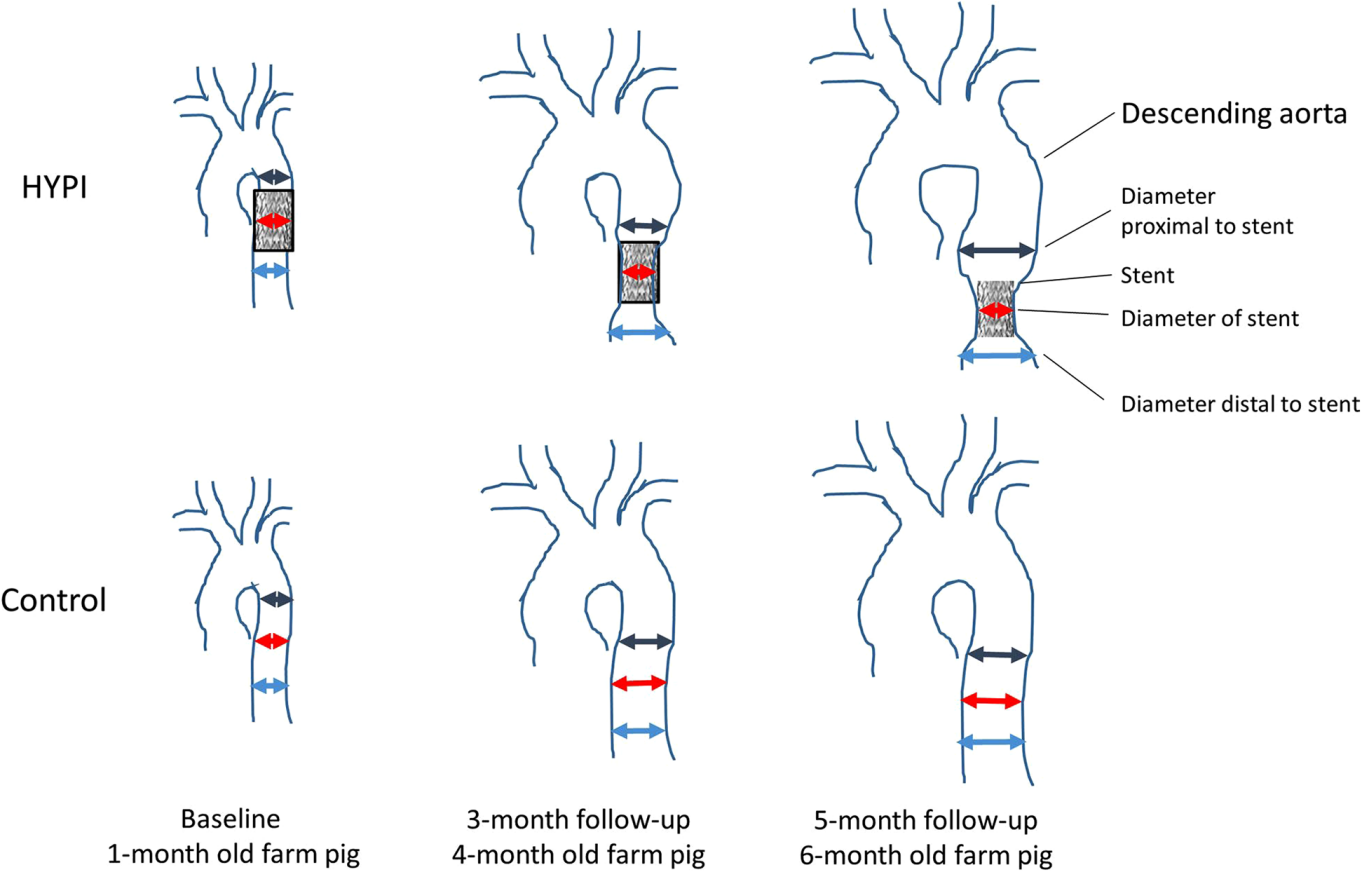
Schematic illustration of the development of artificial aortic isthmus stenosis. HYPI: group of cardiac hypertrophies. From (52).

An aortic stent has effects similar to aortic banding but is less invasive because it requires a percutaneous procedure instead of open-chest surgery. The stent is ingrown into the aortic wall and does not allow normal aortic growth due to the stent's constant size, which causes partial antegrade obstruction and stenosis in the aorta (Figure 5). To date, this technique has been used in only one study (52) in which a pig showed significantly changed diastolic function and LV morphology, increased BNP, and pulmonary hypertension. The HFA-PEFF and H2FPEF scores were 6 and 3, respectively (Table 2). Pathology showed LV fibrosis and hypertrophy.

**Table 2:** Clinical scores of aortic stent model.

		HFA-PEFF				H2FPEF								
Article	Species	Function	Morphology	BNP	Total	Obesity	HT	Age	AFib	PH	DD	Total	Gold standard	EF preserve
Gyöngyösi et al. (52)	Pig	2	2	2	6	0	1	0	0	1	1	3	Yes	Yes

AFib, atrial fibrillation; BNP, brain natriuretic peptide; DD, diastolic dysfunction; HT, hypertension; PH, pulmonary hypertension.

### 5.3. Renal wrap

Renal wrap has been used primarily in dogs. Briefly, the procedure includes performing a midline abdominal incision, followed by wrapping a single kidney or both kidneys through silk or sterilized cellophane without constricting the renal vessels. This method produces persistent renal hypertension due to perinephritis. The hypertension causes LV pressure overload and induces LV hypertrophy. Because dogs can reach “old” age more easily than other animal models, these studies may be useful for examining age as a factor. For example, Munagla et al. (53) wrapped both kidneys with silk tissue and compared old dogs with old and young control dogs. They evaluated diastolic function through catheters but did not perform any echocardiographic evaluation. Their results showed impaired LV diastolic function and increased LV mass with persistent hypertension in the experimental group of old dogs. However, they did not include a young experimental group, and the level of myocardial fibrosis was similar between the experimental group consisting of old dogs and the control group consisting of young dogs; therefore, it was challenging to determine the influence of age in this model. Hamdani et al. (54) used the same methods and groups in dogs and obtained similar results, in addition to observing weight gain in the experimental group of old dogs. No studies included any BNP results. Therefore, under either clinical score system, none of these studies qualified as an HFpEF model (Table 3). Although there are reports of other species, such as pig or nonhuman primates, they do not specifically focus on the HFpEF model; therefore, those reports were not included in this review.

**Table 3:** Clinical scores of renal wrapping models.

		HFA-PEFF				H2FPEF								
Article	Species	Function	Morphology	BNP	Total	Obesity	HT	Age	AFib	PH	DD	Total	Gold standard	EF preserve
Munagla et al. (53)	Dog	2	2	0	4	0	1	1	0	0	1	3	Yes	Yes
Hamdani et al. (54)	Dog	2	2	0	4	1	1	1	0	0	1	4	Yes	Yes

AFib, atrial fibrillation; BNP, brain natriuretic peptide; DD, diastolic dysfunction; HT, hypertension; PH, pulmonary hypertension.

### 5.4. Renal clamp/embolization

For the renal clamping procedure, an adjustable clamp made of pure silver or stainless steel is used, and each clamp is attached to the renal artery at around 1 cm distal from its origin to apply an arbitrary extent of ligation. Many studies have described the use of clamps to induce hypertension in dogs, but none focused on HFpEF. Recently, renal embolization has been used as a new technique to induce hypertension by injecting microspheres through the renal artery to embolize the whole kidney. To date, one study has described renal embolization as one of multiple ways to induce HFpEF. Therefore, this study is discussed in the section “combination models.”

### 5.5. Medication

Deoxycorticosterone acetate (DOCA) is a mineralocorticoid receptor agonist that acts as a precursor to aldosterone. The medication is administered subcutaneously (100 mg/kg) in pellets and is released over 90 days, with the aim of promoting sodium and water retention. One study (55) demonstrated that treating pigs with DOCA increased mean arterial pressure by approximately 20 mmHg compared with the control. In addition, cardiovascular magnetic resonance imaging analysis showed left atrial enlargement, and E' and E/E' markers were the most different between DOCA and control pigs at rest. Inadequately increased myocardial perfusion reserve during stress may represent a metric for early-stage HFpEF; overall, it had a HFA-PEFF score of 4 and a H2FPEF score of 2 (Table 4).

**Table 4:** Clinical scores of DOCA model.

		HFA-PEFF				H2FPEF								
Article	Species	Function	Morphology	BNP	Total	Obesity	HT	Age	AFib	PH	DD	Total	Gold standard	EF preserve
Reiter et al. (55)	Pig	2	2	0	4	0	1	0	0	0	1	2	No	Yes

AFib, atrial fibrillation; BNP, brain natriuretic peptide; DD, diastolic dysfunction; HT, hypertension; PH, pulmonary hypertension.

In most cases, DOCA was combined with other methods to introduce HFpEF, which is discussed in the section “combination models.”

### 5.6. Diet

Diet can be used as a complementary method for inducing metabolic syndrome in heart failure models. Diets can vary according to the aim of the research, but the most common diet is the Western diet (56, 57), which has a high content of salt and fat. If needed, researchers can supplement with high sugar and calories as a method to induce metabolic disorders that mimic the findings in metabolic syndrome. Diet is most effective in small animals. In large animals, diet is frequently associated with other comorbidities that simulate risk factors related to human HF. However, diet alone is not enough to induce effects on diastolic cardiac function in large animal models.

### 5.7. Combination models

Because HFpEF in humans is a highly heterogeneous syndrome, more researchers are looking to develop models that introduce multiple risk factors, such as a high-fat diet, hypertension, and diabetes. These models may accurately mimic multi-system dysfunction that closely represents human pathophysiologic change (Table 5).

**Table 5:** Clinical scores of combination models.

		HFA-PEFF				H2FPEF								
Article	Species	Function	Morphology	BNP	Total	Obesity	HT	Age	AFib	PH	DD	Total	Gold standard	EF preserve
Olver et al. (57)	Pig	2	2	0	4	2	1	0	0	0	1	4	Yes	Yes
Sorop et al. (58)	Pig	2	0	0	2	0	1	0	0	0	1	2	Yes	Yes
Shapiro et al. (59)	Dog	2	0	2	4	0	1	0	0	0	1	2	Yes	Yes
Sharp III et al. (60)	Pig	2	2	2	6	2	1	0	0	1	1	5	Yes	Yes
Zhang et al. (61)	Pig	2	2	2	6	2	1	0	0	1	1	5	Yes	Yes
Schwarzl et al. (62)	Pig	0	2	0	2	0	1	0	0	0	1	1	Yes	Yes
Muhlfeld et al. (63)	Pig	0	0	0	0	0	1	0	0	0	0	1	No	NA

AFib, atrial fibrillation; BNP, brain natriuretic peptide; DD, diastolic dysfunction; HT, hypertension; NA, not applicable; PH, pulmonary hypertension.

Sorop et al. (58) introduced a triple-factor model in pigs that included type 2 diabetes induced by streptozotocin, hypercholesterolemia produced by a high-fat diet, and hypertension produced by renal embolization. In addition to targeted risk factors, the experimental pigs presented systemic inflammation and diastolic dysfunction while the EF was maintained in the normal range. However, a high-fat diet did not help the pig to develop obesity; conversely, experimental pigs weighed significantly less than did control pigs, which the authors attributed to growth retardation resulting from renal dysfunction. Therefore, this combination model did not result in a high clinical score.

In another combination model in which multiple methods were used to induce risk factors, renal wrap and DOCA were performed in old dogs (59). At the end of the experiment, these dogs showed significantly elevated blood pressure and deterioration of diastolic function compared with dogs treated only with renal wrap. However, the LV mass between groups was not significantly different. In this study, echocardiography and hemodynamic studies were performed only in the final week. Therefore, the lack of baseline and healthy control data limit the probability that the LV morphology change observed qualified as HFpEF.

The combination of diet and hypertension—the most common multiple comorbidities of HFpEF—has also been applied as a model. In a study of mini-pigs fed a diet high in fat, fructose, and salt combined with DOCA, the pigs developed obesity, hypertension, and diastolic dysfunction with pulmonary hypertension (60). This model demonstrated comprehensive systemic changes. In another model described by Zhang et al. (61), pigs were given a combination of DOCA, angiotensin II (to produce hypertension), and a Western diet. The experimental pigs showed significant changes in diastolic dysfunction, LV hypertrophy, obesity, pulmonary hypertension, and BNP levels. In a study of Ossabaw pigs in which the use of aortic banding was combined with a high-fat diet, similar comprehensive systemic responses were observed (57), including hypertension, cardiac hypertrophy, features of diastolic dysfunction from catheter measurements, peripheral and central vascular dysfunction, and obesity with a systemic inflammatory state. Significant changes were also observed in the experimental group's gene pathways related to cardiac and pulmonary fibrosis, pulmonary hypertension, and atrial fibrillation. Furthermore, the dobutamine-induced functional kinetic reserve was examined on the molecular level. However, under the clinical scoring system, this study did not provide the BNP measurements and lacked direct measurements for pulmonary hypertension and atrial fibrillation, resulting in a score that was not as good as others.

In another study, Schwarzl et al. (62) induced hypertension and hyperlipidemia in landrace pigs by using DOCA and a Western diet containing high amounts of salt, fat, cholesterol, and sugar for 12 weeks. Compared with weight-matched controls, pigs treated with DOCA and a Western diet showed LV concentric hypertrophy and left atrial dilatation; however, no change in BNP was detected, and diastolic function changes from echocardiography were lacking, although the authors provided stress data to show decreased heart functional reserve. Mühlfeld et al. (63) induced HFpEF in pigs by treatment with DOCA combined with a high-salt/high-lipid diet for 3 months and compared them with normal weight-matched pigs. The study was focused on histopathology and DOCA-induced changes in cardiomyocytes (diameter, subcellular composition) within all layers of the LV free wall. DOCA induced changes in the interstitium, which appeared to be more pronounced in the subendocardial ventricular wall layers. However, the study lacked functional analysis, resulting in a very low score.

## 6. Discussion

Using the two most common clinical score systems, we evaluated large animal models of HFpEF that are currently used in the research field. When we combined all of the single-factor studies or used the most popular single-factor study of aortic banding to compare with the combination models, we found among the large animal studies that HFA-PEFF criteria were easier to meet than those of the H2FPEF score, whether the study was a single-factor or multiple-factor design (Table 6). For the H2FPEF score, the combination models had better scores, but only 2 of them were close to the threshold as high-probability HFpEF. As we mentioned above, the HFA-PEFF score focuses more on cardiac features, whereas the H2FPEF score has more extra-cardiac factors. Therefore, we believe a combination model with multiple risk factors will be more likely to reach a high H2FPEF score and better reflect the heterogeneity of HFpEF. We also noted that the use of an aortic stent, renal wrapping, and DOCA had better H2FPEF or HFA-PEFF scores. Because the number of studies is limited compared with the number of studies of aortic banding and combination models, it is difficult to conclude whether they are better models. From the overall comparison, the combination models still had the better H2PEF scores. New techniques, such as telemetry medicine, will help to facilitate a multiple-factor model more easily than before. Our group is using a triple-factor model in pigs that includes hypertension, diabetes, and hypercholesterolemia. In our model, we integrate many new techniques, such as the use of a continuous LV pressure sensor, EKG sensor, and blood pressure sensor. We hope these sensors can provide more valuable information when we reach the endpoint.

**Table 6:** Comparison between single-factor models and combination models.

	Number of Studies	Mean HFA-PEFF score	Mean H2PEF score
Single factor models	16	3.8 ± 1.8	2.0 ± 2.8
Aortic banding	12	3.5 ± 1.9	1.8 ± 1.1
Aortic stent	1	6	3
Renal wrapping	2	4	3.5 ± 0.7
DOCA	1	4	2
Combination models	7	3.4 ± 2.2	2.9 ± 1.7

DOCA, deoxycorticosterone acetate.

For the models that received a lower score under the H2FPEF, we believe there are several reasons. First, most models failed to produce obesity, even with a high-fat diet, which is an interesting topic. In our own experience, we realize not every animal has the same appetite for a high-fat diet. Animals may lose weight if given a food that they do not want to eat. Also, some other factors introduced into the model, such as renal dysfunction, may further affect the animals' appetite. Second, many models lacked methods for monitoring. For example, atrial fibrillation is an important factor to consider, and most studies made no mention of EKG. Currently, as telemetry becomes more and more popular, we believe the tool can be used to monitor EKG for a long time in animals. For example, in our own studies, we implant an EKG recorder into the pig's body to provide a continuous EKG monitor. We hope that this allows us to capture the EKG changes during the whole period and make the model more reliable.

Echocardiography plays an important role in both of the score systems studied. Therefore, an experienced echocardiographer is essential for the whole study. Unfortunately, many studies did not provide good echocardiographic evaluations, especially for diastolic function. Although hemodynamic measurement is the gold standard for evaluating diastolic function, it is invasive, complicated, expensive, and lacks repeatability and is thus rarely used in clinical evaluations. Therefore, a method that is consistent with clinical application should be applied to validate the model. For example, echocardiography can be used to monitor the animal weekly by noninvasive means and show a clear trend of diastolic function change. In contrast, hemodynamic measurement can be scheduled only 2 or 3 times during the study period because of its invasiveness. Some authors reported that echocardiography was not good because of the animals’ anatomical variation. In our own experience, at least with pigs, good images can be easily obtained through the subcostal view, with clear mitral valve flow and tissue Doppler signals. Therefore, we strongly suggest that diastolic function data be evaluated by using echocardiography and that hemodynamic measurement be reserved for additional verification.

It is important to note that the clinical scores evaluated in this study do not cover all facets of HFpEF. For example, the scores do not account for pathology, change of peripheral vascular function, or stress/exercise tests, which are valuable for understanding the pathological changes in the disease. Just as in the combination model (57) discussed above, a much wider range of systemic analyses was presented that included peripheral vascular changes and gene pathways, but the clinical score was not high. Therefore, when designing studies, the ultimate goal should not be only to improve clinical scores. Monitoring pathologic change and performing novel gene sequence analysis will help improve our understanding of HFpEF. In other words, although some animal models received lower scores in the clinical score systems, this does not mean that they are not a good model. The purpose of using the clinical score systems is to have some objective criteria by which to judge, especially for evaluating a new medication or device, and to help researchers design a comprehensive model that addresses clinical needs (61).

## 7. Conclusion

HFpEF is a complex disease with multiple contributing comorbidities that make it difficult to develop effective preclinical models. Progress in the field requires agreement on key features of animal models of HFpEF. Two new clinical scores have recently been developed to define these features. With this review, we are the first to our knowledge to propose using these scores to evaluate large animal models of HFpEF and create a checklist for optimizing translational research. Although most animal models do not meet all criteria, some multifactorial models resemble human HFpEF and may be the future of research. Our proposed approach aims to fill major gaps in HFpEF pathophysiology and facilitate the development of new therapeutics.
